# An examination of the structural linkages between households and community health services in realization of accelerated primary healthcare delivery in Kisumu County, Kenya: a systematic review

**DOI:** 10.12688/f1000research.73303.1

**Published:** 2021-10-25

**Authors:** James M. Wakiaga, Reginald Nalugala

**Affiliations:** 1 Institute of Social Transformation, Tangaza University College, Tangaza University College, Nairobi, Kenya

**Keywords:** community health services, community health workers, primary healthcare, Kisumu County, universal health coverage

## Abstract

**Background:** The provision of community health services (CHS) is critical in accelerating primary health care delivery to vulnerable and deprived populations. However, the structural linkages between households and the CHS being provided by community health workers (CHWs) or volunteers in Kisumu County, Kenya, remains weak and inefffective.

**Methods:** This study applied a descriptive approach using a systematic review technique to provide context and substance to the two main research questions: (1) how does the interaction between households and CHWs affect utilization of CHS to promote equity and right to health? (2) How do health-seeking behaviours of households influence their decision-making regarding choices of CHS? We screened the literature from Google scholar, JSTOR, SAGE and EBSCO based on our inclusion criteria, resulting in 21 studies. These studies were assessed for quality and eligibility and data extracted based on relevance to the research study.

**Results:** Households place primacy on trust and confidentiality in the interaction with CHWs and this affects uptake of CHS. The social determinants of health are also critical in influencing the health-seeking behaviour of households and individuals and their choice of CHS. The successful models of CHS share the characteristic of community ownership and participation and provides for comprehensive health care teams.

**Conclusion:** CHS are critical for the acceleration of primary health care delivery. It forms an important pathway for the achievement of universal health coverage, which is an outcome required for Sustainable Development Goal 3 on health.

## Introduction

The provision of community health services (CHS) is critical in accelerating primary health care (PHC) delivery to the most vulnerable and deprived populations. According to the World Health Organization (WHO), ^1^ community health workers (CHWs) have been widely used to deliver key health care and health promotion interventions in under-served populations in resource-limited settings and communities. In fact, *The Alma Ata Declaration* signed in 1978 declared PHC as the official health policy of member states. It defined PHC as ‘essential health care based on practical, scientifically sound and socially acceptable methods and technology made universally accessible to individuals and families in the community through their full participation and at a cost that the community or country can afford to maintain at every stage of their development in the spirit of self-reliance and self- determination’. ^2^

This notion of PHC was further reinforced by the 2008 WHO report entitled ‘PHC: Now, more than Ever’, which underscored the case for universal health coverage (UHC) as an instrument to improve health equity, health systems strengthening, being people-centred and purposed to promote and protect the health of communities. The 2008 report of the WHO Commission on Social Determinants of Health ^3^ underlined that social determinants such as income, education and political conditions of countries and societies are critical for health improvement. ^5^ Today UHC has become a critical policy imperative to advance the ideas of social justice, human rights, and equity.

At the global level, the policy imperative for PHC and the role of community health system is anchored on the *Alma Ata Declaration* and most recently on the 2018 *Astana Declaration* in marking the 40th Anniversary since the Alma Ata. The *Astana Declaration* provides an opportunity to take stock of the global progress in supporting community health systems and to rekindle global interest on PHC and the centrality of community health systems to achieving UHC. Held under the banner “From Alma-Ata towards Universal Health Coverage and the SDGs”, it calls for the strengthening of PHC to be inclusive, effective and efficient to realize a sustainable health system that guarantees the achievement of UHC and health-related Sustainable Development Goals (SDGs).

Agarwal et al ^4^ observe that the Astana Conference provides a shift from whether CHW programmes are effective to identifying what makes the CHW programmes effective. Regretfully, world governments have been slow in formulating national policies, strategies and plans of action to launch and sustain PHC, especially in developing countries, as part of a comprehensive health system. ^3^ Underlining this argument is the notion that the success of PHC will, among others, be driven by empowering individuals and communities through their participation in the development and implementation of policies and plans impacting on health outcomes.

Africa has an enormous task to achieve an inclusive and transformation growth unless it invests in sustainable health systems. The WHO estimates that investing an additional $21 to $36 per capita per year over the next five years in Africa would save 3.1 million lives, out of which 90% would be mothers and children. It would also prevent 3.8 million to 5.1 million children from stunting with anticipated economic gains in five years amounting to $100 billion from additional investments in health. ^53^ According to Rifkin, ^5^ it is estimated that by 2030, nearly 9 out of 10 of the extreme poor will be found in sub-Saharan Africa. The WHO ^6^ gives an even more stark picture, pointing out that while the African region constitutes 11% of the world population, it has 60% of people living with HIV/AIDS and constitutes 300-500 million malaria cases occurring every year globally. The continent faces many other health challenges such as polio, rising cases of non-communicable diseases (NCDs), and high maternal and child mortality rates. Most countries did not meet the health-related Millennium Development Goals (MDGs) by 2015 and are not on track to achieve SDG3 on good health and well-being.

The dwindling fiscal space for health financing in Africa has further accentuated the challenge of meeting the health goals. Most countries are failing to meet the 2001 Abuja Declaration target of allocating 15% of total government expenditure to the health sector, and only five countries (Botswana, Rwanda, Zambia, Madagascar and Togo), have met the Abuja target so far. ^53^ Data from the World Bank ^7^ shows a significant increase in out-of-pocket expenses in almost all countries, and the regional average peaked from US$15 per capita in 1995, to US$38 in 2014. This translates to 11 million Africans falling into poverty every year due to high out-of-pocket payments. The fragility of Africa’s health system provides for a policy imperative to strengthen CHS to give impetus to the role of CHWs to accelerate PHC delivery and reduce disease burden. The Third Global Forum on Human Resources held in 2013 recognized that CHWs programmes could play a critical role in accelerating MDGs/SDGs and achieving UHC. ^8^ The WHO Africa region has argued the case for increased scale up of access to PHC services and progress towards UHC by promoting the expansion and implementation of CHW programmes. ^8^ This position is in fact a build up to the 1987 Bamako Initiative adopted by the African Ministries of Health on increasing access to essential drugs and other health care services by strengthening the PHC services ^9^ and that of the African Union of deploying 2 million CHWs across sub-Saharan Africa. ^10^

In Kenya, both the 2010 Constitution and the Vision 2030 policy blueprint commit to improving the health of the population by declaring health as a basic human right, and have now devolved health services to the counties. In addition, The Kenya Health Policy (2014-2030) seeks to achieve UHC by 2030. ^11^ Despite this policy articulation and existence of robust strategies, the country is far from achieving the health goals. Available data, show that maternal mortality rate remains as high at 362 per 100,000 births, malaria incidence per 1000 population stood at 225, while infant and under-5 mortality remains high at 39 per 1000 live births and 52 per 1000 live births, respectively. Kenya is also grappling with communicable diseases such as HIV/AIDS and tuberculosis, with new HIV infections per 1000 infected estimated at 146 despite a drop in prevalence rate from 6.7% in 2003 to 5.9% in 2015. ^12^ Tuberculosis incidence per 1000 population stood at 90 in 2015. NCDs are increasingly becoming a burden to the health system with rising cases of cancer causing an estimated 21,000 deaths annually. ^13^

To improve health outcomes, especially for poor and vulnerable populations, the Government of Kenya prioritized a community health strategy. The second National Health Sector Strategic Plan (NHSSP II) (2005-2010) adopted the Kenya Essential Package for Health (KEPH), which underlined the policy imperative for community health. The KEPH was operationalized in 2006 as a package for the community health strategy – “Taking the Kenya Essential Package for Health to the Community: A Strategy for the delivery of Level One Services” and further revised in 2014 into the current Community Health Strategy (2014-19). The community strategy is geared towards enhancing community access to health care as an intervention to improve productivity and address poverty in Kenya. The communities have the responsibility to manage their own health while PHC provides the basis for CHS as a fundamental human right, social justice and equity. The implementation of the CHS in Kenya has been less than optimal and the CHWs and volunteers who form the bulk of the workforce for the level one services have weak linkages with households/wards and communities.

Most written studies on challenges facing the CHS have narrowly focused on skill development for CHWs. Therefore, this research study looks at some of the functional and behavioural gaps, the structural linkages between households and CHS delivered by the CHWs, the health-seeking behaviour of the households demanding the CHS, and examines some of the successful community health models with a view to proposing a functional model of community health for PHC delivery in Kenya.

This systematic review is part my Doctorate thesis that focusses on Kisumu county, which has been selected due to its heavy disease burden as well as progress made in the roll-out of CHS. Available data from Kisumu County profile ^54^ shows some progress, albeit slowly, in infant mortality at 54 per 1000 live births, maternal mortality rate of 495 per 100,000 births and under-5 mortality rate of 79 per 1000 live births. Communicable diseases such as HIV/AIDS and tuberculosis remain a major challenge to the health with HIV prevalence rate of 14.6%. The Kisumu County Health Sector Strategic and Investment Plan (KCHSSIP) (2013-2017) forms the main framework/strategy for the roll-out of the community health services under which the community health volunteers (CHVs) and community health extension workers (CHEWs) play an important role as intermediaries of CHS. However, the community health strategy model has been hampered by the weak linkages between households, village and community healthcare systems, which is key to accelerating PHC. The study therefore seeks to examine this challenge and has selected two main questions as follows:

(a) How does the interaction between households and CHWs affect utilization of CHS to promote equity and right to health in Kisumu County?
(b) How do health-seeking behaviours of households influence their decision-making regarding choices of CHS offered in Kisumu County?

### Theoretical approach

The health equity, social justice and Sen’s human capability approach constitute the main theoretical underpinnings of the study, as well as for the guiding questions. The health equity theory underlines the prevailing differences in the quality of health and healthcare across different populations. It is about fair and just opportunities to accessing health by eliminating disparities that impact on health outcomes. For the two major proponents of health equity theory, Black ^14^ looked at two primary mechanisms to explain how the social determinants influence health: cultural/behavioural and materialist/structuralist. The materialist/structuralist explanation looks at people's material living conditions and explains how social determinants influence health. Whitehead ^15^ expanded on the concepts and principles of equity and health asserts that different social groups have differences in health where the disadvantaged group suffer a heavier burden of diseases.

Amartya Sen’s theory of development that views *development as freedom* using the capability approach considers health equity as a matter of social justice. Sen et al ^16^^,^^17^ posits that health equity is about broader issues of fairness and justice that pays attention to the role of health in human life and freedom. At the centre of the health equity theory is the idea that access to health is a right and a matter of social justice for the poor and the vulnerable segment of the population who need to be empowered and build their capability. This injustice can be seen from limited opportunity to achieve health outcomes arising from the social arrangements. Rasanathan et al ^18^ observes that the focus on health inequities has renewed interest in PHC and the social determinants of health asserting that by ignoring the social determinants of health will be exacerbating health inequity.

The study also considers Rawl’s theory (1971) of social justice by espousing the theory of social justice as a framework for fairness and distributive justice that permeates the basic structure of society. ^19^ The theory perceives justice as the first virtue of social institutions and that it must percolate at all levels of society. Hence, access to health must be seen as social justice to the citizens and, as Rawl would argue, ‘the rights secured by justice are not subject to political bargaining or to the calculus of social interests’. ^19^ This theory postulates that the primary concern of justice is the functioning of social structure in a way that major institutions proffer fundamental rights and duties and the privilege and the division of advantages through social interactions.

In summary, health equity must be viewed as a plausible theoretical framework that encompass the principles of social justice, human rights (choice), participation and capability approach through empowerment of the people to make rational choices. This means viewing health equity and social justice as central to community empowerment for health promotion. ^20^ The Rawlsian theory of justice provides a good framework for assessing social inequalities in health that are rooted in the society’s socio-economic institutions and this ties with Sen’s idea of capable institutions. Even though some authors ^16^^–^^21^ critiques of Rawl’s distributive approach to social justice have underlined that capabilities are more relevant on justice matters and provide for a more normative conception of social justice.

## Methods

The systematic review targets both qualitative and quantitative conceptual and empirical studies. The study applies a descriptive approach by using narrative review ^22^ to provide context and substance to the two main research questions:

(1) How does the interaction between households and CHWs affect utilization of CHS to promote equity and right to health in Kisumu County?
(2) How do health-seeking behaviours of households influence their decision-making regarding choices of CHS offered in Kisumu County?

For purposes of searching the literature, the study used the operational terms “CHW/V” defined as ‘members of the community selected from the area with the task of improving the community’s health and well-being and linking the people to primary care services’ and “CHS” operationally defined as ‘provision of community healthcare services to a client or patient usually at home or residential setting’.

### Search strategy and selection

The literature search involved screening articles from peer reviewed journals mainly targeting the year 2009 to the present. Exceptional cases were the historical literature. The following databases were used: Google Scholar, JSTOR, Kenya National Bureau of Statistics (KNBS) register, Kisumu County website, SAGE publications and EBSCO. The following search terms were used: “CHS”, “CHW/V”, “PHC”, “UHC” and combined with the terms used in the main research questions such as “CHW models”, “health equity”, “health-seeking behaviour”, “households and choice of community health services” by using the Boolean operators “AND” or “OR” accordingly. ^23^ Zotero software version 5.0.89 was used to collect and collate the data and to assist in the citation and referencing.

### Inclusion and exclusion criteria

Both conceptual and empirical studies were selected based on the title and abstract bearing relevance to the central role of the “CHW/Vs” and the relationship between households and CHWs, as well as the health-seeking behaviour of the households and how this influences their choice and decision-making on use of community health services provide by the CHW/Vs. These were mainly published studies and ranged from global, regional or Africa in particular, and then Kenya, accordingly, using the funnel approach. Studies not pertinent to the two research questions were excluded. The selection was confined to studies published in English language and the papers were screened by the main Author and then independently reviewed by the co-author based on the criteria of (i) relevance and applicability to the themes; and (ii) consistency of the results.

### Data extraction and analysis

The data was extracted using the narrative analysis synthesis approach and clustered based on the thematic areas of the research questions. This is a technique used to identify, evaluate and then synthesize the available empirical evidence. ^55^ The papers were clustered based on the research questions and using the funnel approach by systematically looking at studies that are global, from Africa, and then Kenya, and the relevance of the content to the themes of the research questions. The quality assessment was done by categorizing the screened papers into the two thematic areas of the research questions. The authors gauged the strength of the empirical evidence adduced and absence of bias using the criteria listed in Table 1. This enabled the authors to independently rate the papers as high, medium or low and settling on the 21 selected papers.

**Table 1.** Summary of conceptual and empirical studies included in the review.

Authors, year (reference)	Setting/Location	Thematic issue	Methods	Linkages between households/community and CHWs influenced by relationship, health-seeking behaviour and community health model	Findings
**Adongo & Asaarik, 2018** ^**40**^	Ghana	Health-seeking behaviour of households	Qualitative	Established choice of treatment method associated with household income, distance to health care facility occupation and education status.	Choice of treatment method associated with socio-economic and geographical factors.
**Aseyo et al, 2018** ^**43**^	Kisumu, Kenya	Relationship between CHW/Vs and Households/community	Mixed Method (Observatory study)	How the households and CHWs correlate determine the quality of services.	Relationship determined by ability of CHWs to provide material support.
**Akeju et al, 2016** ^**32**^	Nigeria	Health-seeking behaviour	Qualitative (Ethnographic)	Choice of services offered by CHW influenced by cultural factors especially on maternal health.	Social determinants of health influenced uptake of CHS.
**Assefa et al, 2019** ^**26**^	Ethiopia	Relationship between CHW/Vs and Households/community	Qualitative	Social determinants of health influence the uptake of CHS.	A positive correlation between community/households and CHWs in the uptake of CHS.
**Druetz et al, 2015** ^**45**^	Burkina Faso	Health-seeking behaviour	Quantitative (panel data)	Lack of awareness of CHW services affected uptake and utilization of CHS.	Low uptake and utilization of CHS due to lack of awareness.
**Grundy & Annear, 2010** ^**29**^	South Africa	Relationship between CHW/Vs and Households/community	Qualitative	Examined how trust and confidentiality enhanced the uptake of maternal health services in KwaZulu- Natal region.	Lack of trust and confidentiality act as a barrier to use of CHWs; Women prefer female CHWs for maternal services.
**Hussain et al, 2019** ^**34**^	Pakistan	Health-seeking behaviour	Qualitative	Direct relationship between utilization of health services and its effect on population and health-seeking behaviour.	Addressing geographical access, socio-economic factors, education and culture will lead to improved health-seeking behaviour and utilization of health facilities.
**Jobson et al, 2020** ^**49**^	South Africa	Relationship between CHW/Vs and Households/community	Qualitative		
**Kok et al, 2015** ^**25**^		Relationship between CHW/Vs and Households/community	Qualitative	Community attitudes influence care-seeking and health-related behaviour.	Lack of essential services and referral facility hamper use of CHS.
**Liverani et al, 2017** ^**41**^	Vietnam	Relationship between CHW/Vs and Households/community	Qualitative	Credibility and utilization of CHW program depend and influenced by perception of households/community.	Trust and confidentiality is key in the uptake of CHS.
**Mazzi et al, 2019** ^**42**^	Uganda	Health-seeking behaviour	Mixed Method (cross-sectional)	Households choice of CHW dictated by trust, relationship-building, proximity and access to health facilities.	Trust is an important determinant on health-seeking and uptake of CHS.
**Mishra, 2014** ^**31**^	India	Relationship between CHW/Vs and Households/community	Qualitative (Ethnography)	Importance of trust, bonding, and social capital in forging linkages between community/households and CHWs.	Trust and relationship-building is critical in the uptake of CHS.
**Mushtaq et al, 2020** ^**35**^	Pakistan	Health-seeking behaviour		Cultural bias for women limits their freedom to make health choices and decisions.	Women lack freedom to make health choices due to patriarchal cultural bias
**Mwendwa, 2018** ^**44**^	Uganda	Health-seeking behaviour	Qualitative	Households attach value to CHWs work but lack of information and ability to support materially is a gap.	Households’ demand for CHS affected by lack of material support.
**N’Gbichi et al, 2019** ^**36**^	Kenya	Health-seeking behaviour	Qualitative	Women prefer home delivery ad culture act as a disincentive to us of hospital facilities to avoid male nurses or doctors.	Cultural bias leads to use of unskilled delivery and avoidance of health facilities.
**Omeire, 2017** ^**39**^	Nigeria	Health-seeking behaviour	Qualitative	Established socio-economic and cultural contexts influence health-seeking behaviour.	Imperative of socio-economic contexts.
**Owek et al, 2017** ^**30**^		Health-seeking behaviour	Qualitative	Established negative perception on breach of confidentiality by CHWs.	Confidentiality is an imperative for uptake of CHS.
**Rachlis et al, 2016** ^**28**^	Kenya	Relationship between CHW/Vs and Households/community	Qualitative	Breach of confidentiality broke linkages between households/community and CHWs.	Confidentiality determines the choice of utilizing the CHWs.
**Shaikh & Hatcher, 2004** ^**37**^	Pakistan	Health-seeking behaviour	Qualitative	How social determinants of health (socio-cultural) influence utilization of PHC.	Social determinants of health affects utilization of health services

## Results

**Figure 1.**
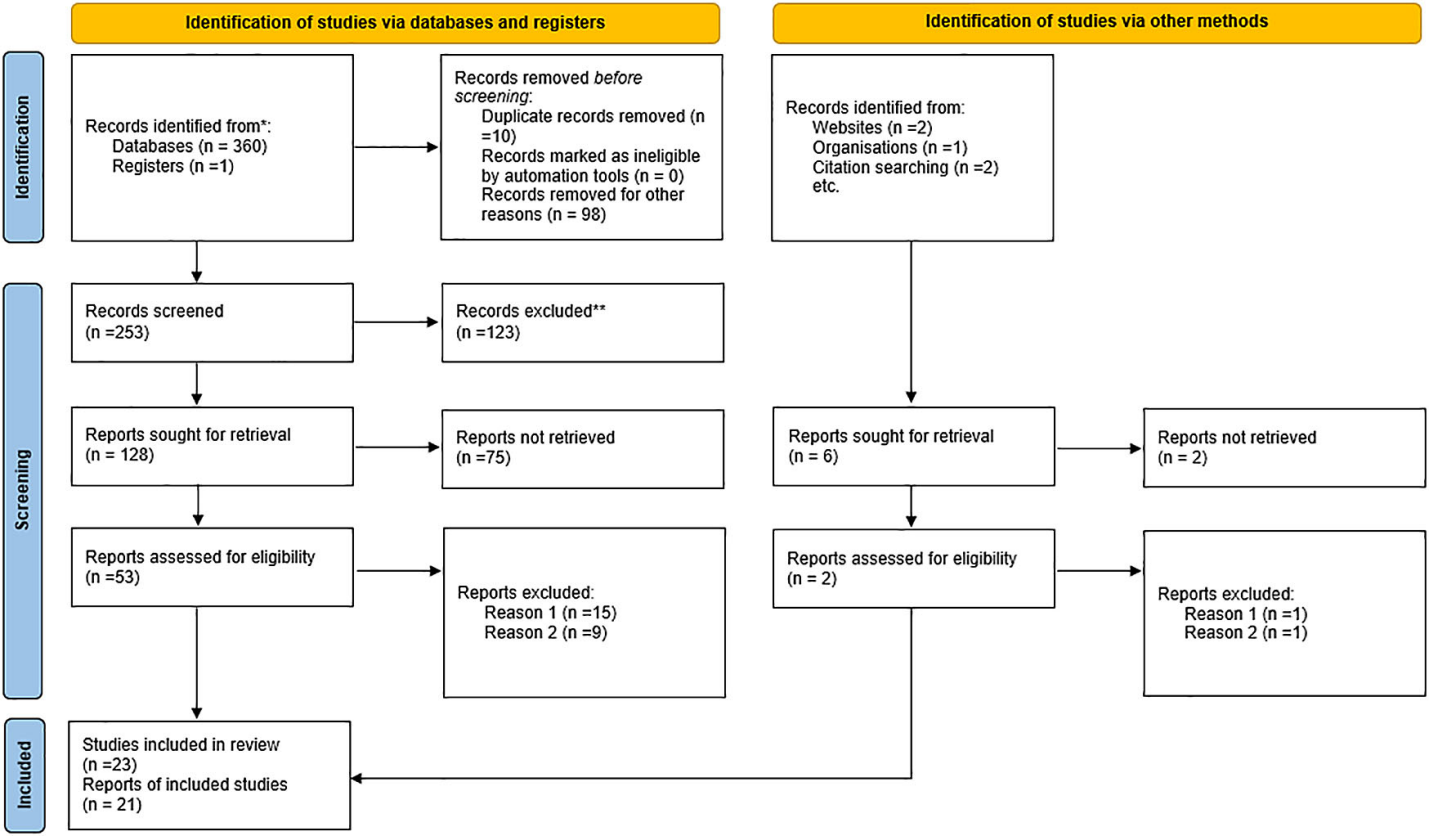
PRISMA flow diagram.

The process of the selection of the studies involved an electronic search identified 366 records, from which 21 studies were retained ( Figure 1). The 21 studies included 19 qualitative and 1 mixed-methods and one randomised control trial. The studies were conducted in India, Cambodia, Uganda, Ethiopia, Burkina Faso, South Africa, Ghana and Kenya. Table 1 lists all studies included in this study.

The quality of the studies was good in terms of providing evidence and application of the methods though the authors offer a critique in terms of relevance to the research questions to determine a research gap. Hence it is important to underline that most of these studies have been narrow in scope and limited to analysing the role of CHWs in the health system and less on community and systemic issues that impact on their performance. The studies have also not focused on the contextual relations between the health volunteers and communities they serve especially from the perspective of the recipient of the services in this case the households. ^24^

### Interaction between households/wards and CHWs and effect on utilization of CHS

Findings from the narrative synthesis of the systematic review of the study shows that the issue of community/household perception of the CHWs as an emerging thematic factor. Most of the studies have cited how the community perceives the CHWs has a positive correlation to the uptake of the CHS. Kok et al ^25^ has identified three areas as key to the effectiveness of CHS: attitudes of the health personnel and communities towards CHWs, the management and structure of health systems and resource allocation guided by the principle of equity, and the quality of community participation. Hence forging a stronger linkage between CHWs and households/community will improve the uptake of the CHS and contribute to accelerated PHC delivery for poor and vulnerable populations.

The quality of interaction between households/communities and the CHWs impact on the effectiveness of the CHW interventions. Kok et al ^25^ posits that policymakers of CHW interventions must take into consideration the socio-cultural, economic and political contextual matters when designing the programmes in order to optimize performance. Two studies ^25^^,^^26^ have indicated that the socio-cultural factors influence the perceptions and relationship between CHWs and households or community. Other pertinent factors influencing the interaction is lack of alternatives to health service delivery that caters for the disadvantaged population and the dissatisfaction of CHWs that may arise due to lack of essential medicines and limited referrals. ^25^^,^^27^

One of the selected studies also considers at length the effectiveness of community structures, such as the village/ward committees or community organizations specifically on leadership capacities and participation of interest groups. For instance, people living with disabilities or women are in some cases not represented in these committees responsible for decision-making on CHS. The studies selected have also cited several other factors that influence this relationship/interaction including the lack of confidence in CHWs, lack of relationship-building with households/community, inability of communes/communities to provide resources to support the work of the CHWs, lack of community mobilization skills and misunderstanding on the role of the CHWs by the community. ^28^

An emerging theme from the selected studies is the importance of trust and confidentiality in improving interactions between CHWs and the household/community. Studies have cited examples from Philippines, Brazil, India and South Africa ^28^^–^^30^ and all demonstrate that issues of trust, bonding, social capital and relationship-building as paramount in establishing linkages with communities and enhancing the uptake of CHS. ^31^

### Health-seeking behaviour of households and the choice of CHS

The selected studies at global, regional and local levels based on the narrative synthesis review have highlighted how the health-seeking behaviour influences the choices of households/communities in utilizing the CHS provided by the CHWs. ^32^^–^^38^ Findings show that a total of seven studies focussing on the role of social and cultural factors shows the impact on the choice of CHS from detection of illness to choosing the health care services. ^39^ In Pakistan, Mushtaq et al ^35^ found that the low position of women from a cultural perspective undermines their freedom to make health choices for themselves and their children without the consent of the head of household. In some cases, the limited number of female health workers limits the women’s access to health care and influences their decision on whether to deliver children at home or in a facility using skilled personnel. ^32^^,^^35^^,^^36^

Three key selected studies looked at geographical factors and how distance from the health facility could influence the health-seeking behaviour and the choice of CHS. ^40^^–^^42^ For instance, the study by Mazzi et al, ^42^ conducted in Uganda, examined the geographical factors that influence health-seeking behaviour by looking at proximity to the health care services. Even though this study could not explicitly explain whether the effect of bringing health facilities closer to the people would make them prefer seeking primary health care directly and avoid CHWs. ^42^

The issues of trust and confidentiality in the interaction between households/community and CHWs equally featured when it comes to the health-seeking behaviour. Some of the selected studies explored how trust influences the health-seeking behaviour and towards selection of CHW as a first point of service. A study by Akeju et al ^32^ in Ogun State, Nigeria, revealed that women utilized multiple caregivers during pregnancy, which was influenced by entrenched trust in traditional birth attendants who live among the community and have established trusted relationships. Studies also show that in Kenya choice of CHWs for PHC support in most disadvantaged households was hinged on trust/confidentiality. For example, mothers in rural Kenya trusted that the CHVs could increase their knowledge of maternal and newborn health. ^33^ The studies analysed reinforced the idea that most qualitative studies on the impact of CHW programmes have narrowly focused on direct CHW management and less attention to how clients or beneficiary communities of the CHW programmes perceive services provided by CHW/Vs as part of the structural linkage of optimizing the CHS to vulnerable populations. ^24^

## Discussion

This systematic review has explored the structural linkages between community/households and the CHS by examining some select studies from the global, regional and local level to understand the interaction between households and CHWs. The systematic review has examined two dimensions that affect this linkage in terms of how the interaction between households and CHWs affect the uptake of CHS and how the health-seeking behaviour of households influence their choices of CHS being delivered by CHWs in the acceleration of PHC services. Evidence from the 21 selected studies show that improving this structural linkage is critical for the uptake of the CHS and towards subsequent acceleration of PHC delivery. The achievement of the SDG3 on good health, and well-being target 3.8 to achieve UHC will depend on the acceleration of PHC for poor and vulnerable populations.

Evidence from the studies show that the relationship between household/communities and CHWs is important in strengthening the interaction and optimize the utilization of CHS. ^31^ Important factors that impact on this relationship includes the social determinants of health, socio-cultural influences, importance of trust and confidentiality, and building on the social capital. A study conducted in Odisha, India, for example, observed that trust-building with the community is a critical determinant on utilization of CHS. This was also corroborated by the outcome of similar studies conducted in Kenya, Malawi, Mozambique and Ethiopia by Kok et al ^46^ on factors shaping the relationship between households/community, CHWs and the health sector. These authors observed that this relationship is particularly important since CHWs act as intermediaries with the health facilities given their understanding and familiarity of the socio-cultural context. The findings from this particular empirical study re-affirms the importance in the trusting relationship for CHW/Vs as key to performance and also establishes that the bond of relationship between CHW/Vs and their supervisors’ impact on their relationship with the communities.

Trust and confidentiality are very important in fostering strong linkage between the households/community and CHWs and this has an impact on the uptake of the community health services. Most of the selected studies underlined the role of perception and attitudes that influence the interaction. This is corroborated in the study by Rachlis et al ^28^ on community perceptions of CHWs specifically for HIV, tuberculosis and hypertension patients in western Kenya. Their findings showed that some participants’ perceptions of CHWs act as an impediment in the management of chronic diseases, especially issues related to lack of confidentiality or information/knowledge on the subject matter. A study by Grundy & Annear ^29^ in Kwa-Zulu-Natal, South Africa, on the role of CHWs in delivering maternal and child health services, observed that the lack of trust and confidentiality was perceived to be the most singular barrier to CHW acceptability and those CHWs with reputation of confidentiality were trusted by the individuals, households and communities. Evidence from the studies also observed that the interaction between the households and CHWs was in some instances influenced by the ability or inability to provide extra resources, especially to needy households. Some poor households see the CHWs not just as a medical aide support but that they should be able to meet their financial needs. ^27^^,^^43^^,^^44^

This systematic review of the studies shows that health-seeking behaviour of household and communities influence their health care preferences. ^34^ This is important for the poor and vulnerable communities who suffer from catastrophic health expenditure and rely on CHWs as their best preference. Using the Pathways model developed by Ref. 38, the social and cultural factors affect the steps of the process from the detection of symptoms to choosing health care services. A study by Mushtaq et al ^35^ in Pakistan provides evidence to show that issues of gender and NCD burden are critical in influencing the health-seeking patterns of households.

Studies are also showing that women are disadvantaged due to cultural reasons from making health choices for themselves and their children without the consent of the head of household. ^32^^,^^35^ In some contexts, the limited number of female health workers limits the women’s access to health care and accounts for the growing burden of the NCDs, such as hypertension. This is also corroborated in a qualitative study conducted in North Eastern Kenya by N’Gbichi et al, ^36^ which observed that women will prefer home-delivery by a skilled attendant if there are no female nurses to attend to them in a hospital facility. This buttresses the point that while delivery in a health facility is the preferred choice, culture could act as a disincentive against using the facility and home delivery is preferred to avoid male nurses or doctors.

The households/community perception on issues of trust and confidentiality does affect the choices they make on whether to utilize the CHWs. ^41^ Other factors that influence the uptake of the CHS include distance to reach communities, infrastructure of geographical location, credibility, lack of awareness of the CHW services or poor engagement amongst the community. ^41^^,^^45^

### Implications for community health models

This systematic review has demonstrated that fostering sustainable linkage between households and community health services provided by the CHWs programmes is key to optimal functioning of the community health strategies and models. This is also in line with the *Alma Ata Declaration* that has been implementing community health strategy to accelerate the delivery of PHC and achieve UHC. This means putting in place an integrated system that has the people and community at the centre of the governance structure of health models to accelerate PHC delivery. Bitton et al ^47^ posits that in most low and middle-income countries, what individuals and community receive from the community health models is not on par with effectiveness and care delivered.

Based on the successful experience of developed models like Brazil’s FHS and Ethiopia’s Health Extension Programme, there is no “one-size-fits-all” approach and implementation has to be driven by local context-specific ways that respond to the socio-economic and political realities as well as health system imperatives. ^48^ For example, in a study of contextual factors affecting integration of CHW into the health system in Limpopo, South Africa, Jobson et al ^49^ identified six critical contexts: geographical context in terms of distance between PHC facility and households, socio-economic context with regard to high levels of poverty in Limpopo in relation to ill-health, community context associated with HIV stigma, cultural beliefs, local governance as a supportive role and organization contexts in the form of competitive interests between national health officials, NGOs, operational environment for CHWs, and leadership challenges.

Studies show that Kenya has undertaken the implementation of the community health strategy in the different national counties with varying results. In pastoral nomadic areas of Northern Kenya, findings show a high cost of attrition for CHVs, per capita coverage by CHVs across the different geographical contexts due to population density, livelihoods opportunity cost and benefit, and the social opportunity cost. ^50^ In Mwingi district, while the model was successful in providing maternal and child health services, socio-cultural and economic factors impeded on the progress. ^51^ Kisumu county which forms case study of this research paper has identified weak linkages between households, village and community healthcare systems, which is key to accelerating primary healthcare. Other findings cited the low participation of the community during the program design, recruitment and implementation and hence the need to enhance linkages between community, CHEWs and CHWs right from the onset. ^52^

## Conclusions

Community health services are critical for the acceleration of PHC delivery amongst vulnerable and deprived populations. It forms an important pathway for the achievement of UHC, which is an outcome of SDG3 on health. Fostering strong structural linkages between the households/wards and CHS being provided by the CHWs/Vs would therefore accelerate the achievement PHC delivery.

The findings from this systematic review study have demonstrated that PHC delivery is critical for better health outcomes and to achieve UHC. The studies have revealed that CHS could significantly contribute to the acceleration of PHC and the agency role of CHW/Vs is paramount at a community level. There is an imperative for strengthening the linkages between households/community and CHW/Vs to foster the uptake of CHS. Evidence from the systematic study pinpoints that understanding the health-seeking behaviour of the households is critical as this influences the choices households make on whether to utilize the health services provided by the CHW/Vs.

## Data availability

### Underlying data

All data underlying the results are available as part of the article and no additional source data are required.

### Reporting guidelines

Figshare: PRISMA-S checklist for ‘An examination of the structural linkages between households and community health services in realization of accelerated primary healthcare delivery in Kisumu County, Kenya: a systematic review’, https://doi.org/10.6084/m9.figshare.16798072. ^56^

Data are available under the terms of the Creative Commons Zero “No rights reserved” data waiver (CC0 1.0 Public domain dedication).
