# Supplementary material for: An examination of the structural linkages between households and community health services in realization of accelerated primary healthcare delivery in Kisumu County, Kenya: a systematic review
Source: F1000Res. 2024 Jun 25;10:1082. Originally published 2021 Oct 25. [Version 3] doi: 10.12688/f1000research.73303.3 (PMC11240083; doi:10.12688/f1000research.73303.3)
Supplement: Peer review history

# Reviewer report: Reviewer response for version 3

**DOI:** 10.5256/f1000research.168156.r295617

**Published:** 2024-07-11

**Authors:** Alexander Laar

**Affiliations:** 1 School of Public Health and Medicine, Faculty for Health and Medicine, The University of Newcastle, Callaghan, New South Wales, Australia; 2 REJ Institute, Tamale, Ghana

My comments have been addressed. I have no further comments.

Are the rationale for, and objectives of, the Systematic Review clearly stated?

Yes

Is the statistical analysis and its interpretation appropriate?

Yes

If this is a Living Systematic Review, is the ‘living’ method appropriate and is the search schedule clearly defined and justified? (‘Living Systematic Review’ or a variation of this term should be included in the title.)

Yes

Are sufficient details of the methods and analysis provided to allow replication by others?

Yes

Are the conclusions drawn adequately supported by the results presented in the review?

Partly

Reviewer Expertise:

Public Health, Health Economics, Digital health, maternal and child health, sexual and reproductive health, qualitative methods, mixed methods, Quality improvement in health.

I confirm that I have read this submission and believe that I have an appropriate level of expertise to confirm that it is of an acceptable scientific standard.

# Reviewer report: Reviewer response for version 2

**DOI:** 10.5256/f1000research.162393.r267203

**Published:** 2024-06-08

**Authors:** Alexander Laar

**Affiliations:** 1 School of Public Health and Medicine, Faculty for Health and Medicine, The University of Newcastle, Callaghan, New South Wales, Australia; 2 REJ Institute, Tamale, Ghana

**Recommendation: Major revision.**

**Abstract**

 Introduction

 -In the second sentence of the introduction, ‘by’ should be inserted between delivery and synthesizing.

 In the last sentence, the authors replace ‘primary research’ with policy

 Methods

 -CHWs need to be written in full and bracketed on first use and abbreviated on subsequent use

 - I suggest, the authors phrase the last sentence to read, Data extracted based on the relevance to the research study and assessed for quality and eligibility criteria.

**Manuscript text**

 Introduction

 -2/1000 liver births, liver should be ‘live’

 -PHC need to be written in full and bracketed on first use and abbreviated on subsequent use

 -In Paragraph 3, the evidence needs to be referenced.

 -At paragraph 6, “is” needed to come before imperative

 -SDGs/MDGs need to be written in full and bracketed on first use and abbreviated on subsequent use

 -In paragraph 8, the second sentence and the last 2 sentences need to be referenced.

 - In paragraph 10, the first sentence needs to be referenced.

**Search strategy and selection**

 -2009 to the present. The authors should consider indicating the present year.

 Inclusion and exclusion criteria

 -In the first paragraph, the word provide, should be provided.

 PRISMA flow diagram.

 The authors should go over the numbers to ensure that their + &- are correct.

 Results

 I don’t know the reason for writing reports section as a discussion. They may consider combining the results with the discussion or report them separately by removing the references from the results section. It will be easier to do this by moving the discussion section with the first paragraph to come before “Interaction between households/wards and CHWs and effect on utilization of CHS”

 -They should also explain how they reported results from quantitative, qualitative and mixed methods studies.

 Discussion

 -The authors should also consider adding a section for the strengths and limitations for the study. They should also consider using the information in paragraph 2 as limitations for the study.

 - They should also consider adding a section for policy implications and future research.

 References

 The references need editing. While the text shows a total of 52, the reference section shows 56 references.

 -the authors need to follow the author guideline in preparation of the manuscript

Are the rationale for, and objectives of, the Systematic Review clearly stated?

Yes

Is the statistical analysis and its interpretation appropriate?

Yes

If this is a Living Systematic Review, is the ‘living’ method appropriate and is the search schedule clearly defined and justified? (‘Living Systematic Review’ or a variation of this term should be included in the title.)

Yes

Are sufficient details of the methods and analysis provided to allow replication by others?

Yes

Are the conclusions drawn adequately supported by the results presented in the review?

Partly

Reviewer Expertise:

Public Health, Health Economics, Digital health, maternal and child health, sexual and reproductive health, qualitative methods, mixed methods, Quality improvement in health.

I confirm that I have read this submission and believe that I have an appropriate level of expertise to confirm that it is of an acceptable scientific standard, however I have significant reservations, as outlined above.

# Response

**Published:** 2024-06-19

**Authors:** James Wakiaga

**Affiliations:** Tangaza University College, Kenya

Thank you for the valuable comments to the article. We have taken on board all the editorial comments and amended as advised. In addition, we have aligned the section on results and Discussion as recommended to ensure it flows well. We have also added a section on strengths and limitations and this adds value to the article. We have also taken on board comments regarding the reporting of the results of qualitative, quantitative and mixed methods and attempted to explain the application of the thematic analysis approach was applied without using any particular metric. This kind of measurement is also a new area  for health sciences deduced from my reading on how we could address this comment.

# Reviewer report: Reviewer response for version 2

**DOI:** 10.5256/f1000research.162393.r246067

**Published:** 2024-03-05

**Authors:** Henry Perry

**Affiliations:** 1 Department of International Health, Johns Hopkins University Bloomberg School of Public Health, Baltimore, USA

I reluctantly approve this revision with one exception. I still think the title is mis-stated. I would prefer “A systematic review of the structural linkages between households and community health services in realization of accelerated primary healthcare delivery: Implications for Kisumu County, Kenya”.

Are the rationale for, and objectives of, the Systematic Review clearly stated?

No

Is the statistical analysis and its interpretation appropriate?

Not applicable

Are sufficient details of the methods and analysis provided to allow replication by others?

No

Are the conclusions drawn adequately supported by the results presented in the review?

No

Reviewer Expertise:

Primary health care, community health, community heath workers

I confirm that I have read this submission and believe that I have an appropriate level of expertise to confirm that it is of an acceptable scientific standard.

# Reviewer report: Reviewer response for version 1

**DOI:** 10.5256/f1000research.76946.r223647

**Published:** 2023-11-27

**Authors:** Beverly Marion Ochieng

**Affiliations:** 1 Tropical Institute of Community Health, Kisumu, Kenya

**REVIEW**

 Title: An examination of the structural linkages between households and community health services in realisation of accelerated primary healthcare delivery in Kisumu County, Kenya: a systematic review

**Major comments:**

 In reference to your topic, it will be advisable to put into context the meaning of structural linkages between households and community health services in your introduction.

**Abstract**

 Keep the abstract as brief as possible, with a precise, to-the-point example on critical issues that need to be highlighted in the abstract

**Background: **the context and purpose of the study;

**Method: **how the study was performed and statistical tests used;

**Results: **the main findings (Focus on Kisumu County)

**Conclusion: **a ****brief summary and potential implications

**“Methods**: This study applied a descriptive approach using a systematic review technique to provide context and substance to the two major research questions:

 (1) How does the interaction between households and CHWs affect the utilisation of CHS to promote equity and the right to health?

 (2) How do health-seeking behaviours of households influence their decision-making regarding choices of CHS? ”

**The review has not answered the stated research questions, especially regarding Kisumu County.**

 “We screened the literature from Google Scholar, JSTOR, SAGE, and EBSCO based on our inclusion criteria, resulting in 21 studies. These studies were assessed for quality and eligibility, and data was extracted based on relevance to the research study."

 *** Unnecessary sentence in the abstract***

**The introduction**

 Help the reader understand the interpretation of structural linkages between households and community health services from your research point of view. The introduction does link to the title of the study and the research question of the paper mostly sounds more like a world review of CHW services as opposed to what is stated in the research title. At what point do we narrow down the area of study?

 What is the statement of the problem with a focus on Kisumu County? What studies have been done based on your research topic on Kisumu County? What are the policy implications in relation to this study?

**Informative policy documents for your reading**

 1) Community health Strategy document

 2) Kisumu Health Strategic Plan

 3) KDHS (Current)

****

**Results: **contradict what is stated as the research questions; no systematic flow.

 The results do not highlight studies that have been done on structural linkages between households and community health services, especially in this study, which begs the question: have we answered the research questions and are we linking our findings to the research topic?

 The highlighted studies are focusing on the different aspects of community health services and not structural linkages, as stated in the research topic 

**Conclusion:** not well thought through, not coherent with the findings, no mention of studies done in Kisumu in terms of structural linkages between households and community health services

 Recommendations based on findings from Kisumu County based on empirical evidence are conspicuously missing, which is obviously not stated in this paper.

Are the rationale for, and objectives of, the Systematic Review clearly stated?

No

Is the statistical analysis and its interpretation appropriate?

Not applicable

Are sufficient details of the methods and analysis provided to allow replication by others?

Partly

Are the conclusions drawn adequately supported by the results presented in the review?

No

Reviewer Expertise:

HEALTH SYSTEMS RESEARCH  & COMMUNITY HEALTH

I confirm that I have read this submission and believe that I have an appropriate level of expertise to state that I do not consider it to be of an acceptable scientific standard, for reasons outlined above.

# Response

**Published:** 2023-12-29

**Authors:** James Wakiaga

**Affiliations:** Tangaza University College, Kenya

The comments are well received and will attempt to respond to some of the issues raised in reviewing the draft. Notably the issue of conceptualizing  "structural linkages" in the introduction, contextualizing the findings based on the  Kisumu study as well as the abstract. Important to note is that this is  systematic review study and focus was mainly on the available literature that respond to the 2 research questions that form the basis of the research study. hence the literature review could not just be confined to evidence derived from peer-reviewed studies conducted  in Kisumu County but also global, regional and at national/local  level. I will submit a revised paper based on the comments and open to any further suggestions.

# Reviewer report: Reviewer response for version 1

**DOI:** 10.5256/f1000research.76946.r147526

**Published:** 2022-09-12

**Authors:** Henry Perry

**Affiliations:** 1 Department of International Health, Johns Hopkins University Bloomberg School of Public Health, Baltimore, USA

**Major comments:**

 I found this article troubling on several counts.

1. The title of the article and the research questions indicate that Kisumu County, Kenya, is the focus of the research, but there is nothing in the findings that pertain directly to Kisumu County. And, there is no description of the community health services in Kisumu County.
2. The authors state that they are interested in examining structural linkages between households and community health services, but they never clearly state what they mean by “structural linkages.” This should be defined at the outset. In fact, much of the literature they identify from their systematic review focuses on “soft” features of community health services (trust, confidentiality), on the context (social, cultural, economic and geographical factors), or the features of the health system (material support available to CHWs, available of essential services, including referral services). None of these findings provide any insights into actual structural linkages between households and the community health system.
3. Most of the Introduction, while interesting, does not clearly relate to the research question that the authors pose.
4. The authors’ principal conclusion (from the Abstract), “CHWs are critical for the acceleration of primary health care delivery” is not supported by the evidence presented in the Results section. In the Conclusions section, the authors state that their findings “have demonstrated that PHC delivery is critical for better health outcomes and to achieve the UHC [Universal Health Coverage].” However, in fact, their findings do not demonstrate this.
5. On p. 8 middle the authors state “…most of these [included] studies have been narrow in scope and limited to analysing the role of CHWs in the health system and less on community and systemic issues that impact their performance.” This raises the question of why in fact these articles were included?

**Minor comments:**

1. There is no mention of to what degree the article selection methodology meets the standard criteria for a systematic review.
2. What is the “funnel approach”?

Are the rationale for, and objectives of, the Systematic Review clearly stated?

No

Is the statistical analysis and its interpretation appropriate?

Not applicable

Are sufficient details of the methods and analysis provided to allow replication by others?

No

Are the conclusions drawn adequately supported by the results presented in the review?

No

Reviewer Expertise:

Primary health care, community health, community heath workers

I confirm that I have read this submission and believe that I have an appropriate level of expertise to state that I do not consider it to be of an acceptable scientific standard, for reasons outlined above.

# Response

**Published:** 2023-12-29

**Authors:** James Wakiaga

**Affiliations:** Tangaza University College, Kenya

I have taken note of the comprehensive comments by the reviewer and especially pertaining to the title of the research paper. The central focus of the study is Kisumu County, Kenya and the systematic review was mainly to draw out the existing  literature review around the structural issues of relationship between the CHVs and households in accelerating primary healthcare delivery, as well as, the health-seeking behaviour of households. The systematic review was partly to draw out the secondary research information that would guide the framing of the primary research. I also agree on the imperative of defining conceptually what "structural linkages" means for purposes of the research study and this will be addressed in the introduction in the revised draft.
